# Patient journey and treatment pattern in myasthenia gravis: real-world data from the Brazilian public health system

**DOI:** 10.1055/s-0045-1811720

**Published:** 2025-10-27

**Authors:** Renata Andrade, Wilson Marques Junior, Luiza Vasconcelos, Paula Pungartnik, Thaís Zamboni Berra, Lucas Vieira Cortez, Daiane Beneduzzi

**Affiliations:** 1 Hospital Metropolitano Oeste Pelópidas Silveira, Real Hospital Português, Divisão de Doenças Raras, Recife PE, Brazil.; 2 Faculdade de Medicina de Ribeirão Preto, Hospital das Clínicas, Ribeirão Preto SP, Brazil.; 3 Astrazeneca, São Paulo SP, Brazil.; 4 IQVIA, Real World Evidence, São Paulo SP, Brazil.

**Keywords:** Myasthenia Gravis, Standard of Care, Epidemiology, Public Health, Immunoglobulins, Database

## Abstract

**Background:**

Myasthenia gravis (MG) is a rare neurological disease and the most common autoimmune disorder, characterized by muscle weakness. In Brazil, there is limited data on these patients within the Brazilian public healthcare system (SUS, from Sistema Único de Saúde, in Portuguese).

**Objective:**

To assess patients with MG in the Brazilian public healthcare system to understand their characteristics, patient journey, and treatment patterns.

**Methods:**

A retrospective observational study using real-world data from SUS to analyze patients with MG from January 2010 to December 2023. Data were extracted from the DATASUS, focusing on the Outpatient Procedure (SIA) and Hospital Admissions (SIH) information systems. Probabilistic record linkage compiled longitudinal patient data, assessing epidemiology, demographics, clinical characteristics, and treatment patterns, including medications, doses, and lines of treatment (LOT).

**Results:**

A total of 13,476 patients with MG were identified. Admissions in healthcare units increased over the years, with 30.4% experiencing exacerbations and 9% crises. The mean age was 45.6 years, with a majority being female (65.4%) and white (53.4%). The case–fatality rate rose from 0.76% in 2011 to 1.90% in 2023. Treatment patterns showed frequent transitions between LOT, indicating constant instability and inadequate symptom control, with azathioprine and pyridostigmine being the most used medications. Some patients started treatment with intravenous immunoglobulin (IVIg), used in combination with other medications, and continued it continuously.

**Conclusion:**

The study highlights the increasing burden of MG on SUS, emphasizing the challenges of disease management and the need for continuous advancements in diagnostic and therapeutic strategies to improve patient outcomes.

## INTRODUCTION

Myasthenia gravis (MG) is a rare neurological disease. However, it is the most common autoimmune disorder affecting the neuromuscular junction. 1 Its prevalence varies geographically and has significantly increased over the years, ranging from 150 to 200 cases per million. This rise is partly due to improvements in recognition, diagnosis, treatment, and an overall increase in life expectancy. 2

This disease is characterized by fluctuating weakness in the voluntary muscles due to impaired neuromuscular transmission. 1 3 4 Symptoms vary among patients depending on the degree of involvement of the striated muscles. 5 The disorder is classified as ocular myasthenia gravis (OMG) or generalized myasthenia gravis (gMG). The ocular variation affects only the extraocular muscles, causing symptoms such as diplopia and palpebral ptosis. In contrast, gMG involves the bulbar muscles, extremities, or axial muscles leading to difficulties in speaking, raising the arms, and gait instability. 6 This has a substantial impact on quality of life and the ability to conduct everyday tasks. 7

More women than men are affected, and MG can occur at any age. However, onset tends to occur at a younger age in women, with peaks around 30 and 50 years, and at an older age in men, between 60 and 89 years. 8 The disease is marked by periods of remission and exacerbations, with some patients experiencing life-threatening myasthenic crises. 9 Despite advances in understanding the pathogenesis as well as therapeutic development, MG negatively impacts quality of life. Patients face a high treatment burden due to long-term immunosuppressant use, which include adverse effects, toxicities, slow onset of response, and the need for intravenous therapies. 10 Additionally, many patients do not respond to conventional therapies, significantly impacting prognosis. 11

In Brazil, there is limited evidence on the number of patients with MG and their characteristics within the public healthcare system. The Brazilian Clinical Protocol and Therapeutic Guidelines 12 for this condition were recently approved, and there was no real-world information on treatment patterns for these patients. This study aimed to assess patients with MG in the Brazilian public healthcare system, to describe their characteristics, patient journey, and treatment patterns.

## METHODS

### Study design and setting

The present is an observational retrospective study using real-world data from the Brazilian Public Healthcare System ( *Sistema Único de Saúde* , SUS) to describe the patients with MG, their journey and treatment patterns from January 1 ^st^ , 2010, to December 31 ^st^ , 2023.

Cases were identified based on data gathered from administrative claim databases from the Brazilian Health System's Department of Information ( *Departamento de Informações do Sistema Único de Saúde* , DATASUS), that is a comprehensive, national, secondary database that is publicly available from the SUS, which virtually covers 100% of the Brazilian population, and approximately 75% of the population rely exclusively on SUS. 13

Data for the present study was extracted from two information systems within DATASUS: the Outpatient Procedures Information Systems (SIA and the Hospital Admissions Information System (SIH. A probabilistic record linkage 14 15 16 was used to compile longitudinal patient data and combine it. This involved a multi-step process, including date of birth, sex, race, city, and ZIP code. Prior to each step, data cleaning and exclusion of nonlinkage data was performed to retain only high-quality claims for the linkage process. This approach enabled an assessment of each patient's historical record, thereby enabling us to assess their journey through the system.

### Study population

The MG cohort included patients with at least one International Classification of Diseases, Tenth Revision (ICD-10) code G70.00 or G70.01 claim from January 2010 to December 2023, and at least 6 months of follow-up (FUP) information, to assess patients' journey after diagnosis. The index date was the first claim with ICD-10 G70.00 or G70.01 in the study period, considering oMG and gMG without making a distinction. Patients' information will be assessed before and after this first claim.

### Study outcomes and analysis

We assessed epidemiology of MG, demographic characteristics (age, sex, ethnicity, and region of residence), treatment patterns, and out- and inpatient admissions. For the outcomes, the total cohort was stratified based on whether patients experienced exacerbation or crisis. Exacerbation was defined as the occurrence of an intravenous immunoglobulin (IVIg) cycle or an MG-related hospitalization lasting 24 hours or more. A crisis was defined as an intensive care unit (ICU) admission.

For epidemiology of MG, incidence was captured based on the first ICD-10 code (index date) reported during the study period. To measure the disease burden on the public healthcare system, the yearly number of in- and outpatient admissions was assessed. Case fatality rate per year was calculated as the in-hospital mortality by total number of hospitalizations multiplied by 100. Time of FUP of each patient was measured as the time since index up to the last date of patient information available at database.

For treatment patterns, we assessed prescribed medications, their doses, and subsequent lines of treatment (LOTs). Each medication used during validity of prescription, including concomitant medications and doses, was considered a LOT ( Table 1 ). A switch in medication or dose was defined by a new prescription within a 3-month gap, indicating a change in LOT. Concomitant medications also counted as a switch in LOT. This timeframe was based on the duration of the Ambulatory Procedure Authorization (APAC) used for medication dispensation.

**Table 1:** Treatment pattern schemes

Scheme	Medications
1	Azathioprine < 200 mg
	Pyridostigmine < 240 mg
2	Azathioprine ≥ 200 - < 300 mg
	Pyridostigmine ≥ 240 to < 420 mg
3	Azathioprine ≥ 300 mg
	Pyridostigmine ≥ 420 mg
4	Cyclosporine < 350 mg
	Mycophenolate
	Cyclophosphamide
5	Cyclosporine ≥ 350 mg
	Rituximab
6	IVIg

Abbreviation: IVIg, intravenous immunoglobulin.

There were six therapy schemes predetermined based on the clinical practice and insights form. 16 17 18 These schemes also considered treatment recommendations from Brazilian Clinical Protocols and Therapeutic Guidelines (PCDT) for MG, 12 which recommend using the lowest necessary dose to control the disease, with the aim of discontinuing medication if symptoms and signs improve. Each scheme specifies patterns of medications and dosages ( Table 1 ). Due to unavailability of data of oral corticosteroids medications in the database, treatment pattern analysis will capture patients in more advanced LOTs.

The more advanced schemes (4–6) may indicate inadequate symptoms control, disease progression or possible refractory disease. It's important to highlight the dosages of azathioprine and pyridostigmine are usually predetermined based on patients' characteristics. A switch in the LOT between schemes occurs when there is a change in one of the medications and dosage, as described in Table 1 . It is important to note that schemes may include concomitant medications. Even though some combinations were not expected, this study captures real world clinical practice.

Additionally, treatment patterns were analyzed only for patients in more advanced stages of the disease, as the database used for this study does not provide data on corticosteroid medications, which are considered the first LOT for these patients.

We analyzed treatment patterns specifically for patients who began treatment with IVIg, to better understand approaches for managing severe cases. To demonstrate movement between schemes and LOTs, a Sankey diagram was developed.

To evaluate dependence on healthcare units, we analyzed out- and inpatient admissions, based on the highest treatment scheme reached. This means that if patients were on an advanced treatment scheme, they had experienced an advanced stage of the disease at some point in the observation period.

## RESULTS

A total of 13,476 patients with MG were identified according to the inclusion criteria, from January 2010 to December 2023. Patients were followed for an average (SD) of 3 (3.1) years. The number of admissions in the public healthcare system increased over the years, and out of the 13,476 patients with MG, 30.4% (n = 4,100) presented exacerbation. This rate also increased over the years, from 133 (3.2%) in 2010 to 1,301 (31.7%) in 2022. The rate for crises was 9% (n = 1.265), which also increasing throughout the years, from 32 (2.5%) in 2010 to 366 (28.9) in 2023 ( Table 2 ).

**Table 2:** Incidence and number of admissions in the healthcare system of MG cases reported for each year of the study period

Year	Incidence (first ICD-10)	Admission to the SUS*				
		Total MG cohort	Exacerbation		Crisis	
			With	Without	With	Without
	N = 13,476	N = 13,476	N = 4,100	N = 9,376	N = 1,265	N = 12,211
2010	228 (1.7%)	228 (0.5%)	133 (3.2%)	95 (1.0%)	32 (2.5%)	196 (1.6%)
2011	1,110 (8.2%)	1,254 (2.6%)	452 (11.0%)	802 (8.6%)	133 (10.5%)	1,121 (9.2%)
2012	850 (6.3%)	1,785 (3.8%)	560 (13.7%)	1,225 (13.1%)	168 (13.3%)	1,617 (13.2%)
2013	813 (6.1%)	2,237 (4.7%)	709 (17.3%)	1,528 (16.3%)	187 (14.8%)	2,050 (16.8%)
2014	791 (5.9%)	2,519 (5.3%)	782 (19.1%)	1,737 (18.5%)	230 (18.2%)	2,289 (18.7%)
2015	876 (6.5%)	2,968 (6.2%)	893 (21.8%)	2,075 (22.1%)	230 (18.2%)	2,738 (22.4%)
2016	840 (6.2%)	3,206 (6.7%)	927 (22.6%)	2,279 (24.3%)	255 (20.2%)	2,951 (24.2%)
2017	1,051 (7.8%)	3,638 (7.7%)	1,066 (26.0%)	2,572 (27.4%)	295 (23.3%)	3,343 (27.4%)
2018	1,089 (8.1%)	4,091 (8.6%)	1,125 (27.4%)	2,966 (31.6%)	319 (25.2%)	3,772 (30.9%)
2019	1,002 (7.4%)	4,217 (8.9%)	1,144 (27.9%)	3,073 (32.8%)	315 (24.9%)	3,902 (32.0%)
2020	848 (6.3%)	4,330 (9.1%)	1,060 (25.9%)	3,270 (34.9%)	260 (20.6%)	4,070 (33.3%)
2021	1,035 (7.7%)	5,038 (10.6%)	1,151 (28.1%)	3,887 (41.5%)	286 (22.6%)	4,752 (38.9%)
2022	1,971 (14.6%)	6,529 (13.7%)	1,301 (31.7%)	5,228 (55.8%)	355 (28.1%)	6,174 (50.6%)
2023	972 (7.2%)	5,474 (11.5%)	1,111 (27.1%)	4,363 (46.5%)	366 (28.9%)	5,108 (41.8%)

Abbreviations: ICD-10, International Classification of Diseases, Tenth Revision; IVIg, intravenous immunoglobulin; MG, myasthenia gravis; SUS, Brazilian public healthcare system.

Notes: *As it is assessed the admission per year, the same patient can be reported in different years and therefore, the percentage may exceed 100. Exacerbation: patients who had an occurrence of immunoglobulin cycle or MG-related hospitalization ≥ 24 hours. Crisis: ICU admission.

The mean (SD) age of patients with MG was 45.6 (18.0) years. Most of them were female (n = 8,812; 65.4%), white (n = 4,542; 53.4%), and lived in the Southeast region of Brazil (n = 6,708; 49.8%). Patients who presented exacerbation and crisis episodes had a mean (SD) age of 42.4 (18.5) and 40.4 (18.9), respectively. The entire cohort had a FUP average (SD) of 3.1 years (3.1). For stratified groups of exacerbation and crisis, the mean (SD) FUP time was slightly lower, at 2.8 (3.3) and 2.7 (3.3) years, respectively ( Table 3 ).

**Table 3:** Demographic characteristics of MG cases according to exacerbation and crises (with *versus* without)

Patients, N (%)		Total cohort	Exacerbation		Crisis	
			With	Without	With	Without
		13,476	4,100 (30.4%)	9,376 (69.6%)	1,265 (9.4%)	12,211 (90.6%)
Age*	**n valid**	**13,476**	**4,100**	**9,376**	**1,265**	**12,211**
	Mean (SD)	45.6 (18.0)	42.4 (18.5)	47.0 (17.3)	40.4 (18.9)	46.2 (17.6)
	Median (min-max)	45.0 (1.0–99.0)	41.0 (1.0–99.0)	47.0 (1.0–99.0)	38.0 (1.0–99.0)	46.0 (1.0–99.0)
	IQR	27	29	26	28	27
**Age group,** **n (%)**	0–10 years	206 (1.5%)	102 (2.5%)	104 (1.1%)	38 (3.0%)	168 (1.4%)
	11–20 years	879 (6.5%)	423 (10.3%)	456 (4.9%)	164 (13.0%)	715 (5.9%)
	21–35 years	3,163 (23.5%)	1,137 (27.7%)	2,026 (21.6%)	380 (30.0%)	2,783 (22.8%)
	36–45 years	2,559 (19.0%)	664 (16.2%)	1,895 (20.2%)	184 (14.5%)	2,375 (19.4%)
	46–60 years	3,526 (26.2%)	900 (22.0%)	2,626 (28.0%)	266 (21.0%)	3,260 (26.7%)
	61–80 years	2,896 (21.5%)	812 (19.8%)	2,084 (22.2%)	222 (17.5%)	2,674 (21.9%)
	> 80	257 (1.9%)	65 (1.6%)	192 (2.0%)	11 (0.9%)	246 (2.0%)
**Sex** **n (%)**	**n valid**	**13,476**	**4,100**	**9,376**	**1,265**	**12,211**
	Female	8,812 (65.4%)	2,739 (66.8%)	6,073 (64.8%)	864 (68.3%)	7,948 (65.1%)
	Male	4,664 (34.6%)	1,361 (33.2%)	3,303 (35.2%)	401 (31.7%)	4,263 (34.9%)
**Ethnicity, years** **n (%)**	**n valid**	**8,512 (100%)**	**2,879 (100%)**	**5,633 (100%)**	**1,082 (100%)**	**7,430 (100%)**
	White	4,542 (53.4%)	1,537 (53.4%)	3,005 (53.3%)	635 (58.7%)	3,907 (52.6%)
	Brown	422 (5.0%)	117 (4.1%)	305 (5.4%)	42 (3.9%)	380 (5.1%)
	Black	3,198 (37.6%)	1,147 (39.8%)	2,051 (36.4%)	395 (36.5%)	2,803 (37.7%)
	Others	350 (4.0)	78 (2.7%)	272 (4.9%)	10 (0.9%)	340 (4.5%)
**Region of residence** **n (%)**	**n valid**	**13,476**	**4,100**	**9,376**	**1,265**	**12,211**
	North	611 (4.5%)	303 (7.4%)	308 (3.3%)	87 (6.9%)	524 (4.3%)
	Northeast	2,475 (18.4%)	837 (20.4%)	1,637 (17.5%)	216 (17.1%)	2,258 (18.5%)
	South	2,671 (19.8%)	739 (18.0%)	1,932 (20.6%)	253 (20.0%)	2,418 (19.8%)
	Southeast	6,708 (49.8%)	1,811 (44.2%)	4,898 (52.2%)	603 (47.7%)	6,106 (50.0%)
	Midwest	1,011 (7.5%)	410 (10.0%)	601 (6.4%)	106 (8.4%)	905 (7.4%)
Follow-up time,** years	**n valid**	**13,476**	**4,100**	**9,376**	**1,265**	**12,211**
	Mean (SD)	3.1 (3.1)	2.8 (3.3)	3.1 (2.9)	2.7 (3.3)	3.1 (3.0)
	Median (min–max)	1.8 (0.0–13.5)	1.4 (0.0–13.4)	2.0 (0.0–13.3)	1.2 (0.0–13.1)	1.8 (0.0–13.4)
	IQR	3.9	4.5	3.7	4.3	3.9

Abbreviations: IQR, interquartile range; IVIg, intravenous immunoglobulin; MG, myasthenia gravis; SUS, Brazilian public healthcare system.

Notes: *The age of patients was calculated from the date of birth and index date. **Time since index date (ICD-10) up to the last date of patient information available at database.

Among hospitalized patients, 68 related deaths were reported, with most of them occurring in 2021 (n = 14). Across the years, the annual case-fatality rate (CFR) increased from 0.76% in 2011 to 1.90% in 2023. This rate was frequently higher among patients without crisis, and patients without exacerbation did not report any deaths. Among patients with crisis, the CFR ranged from 0.85% in 2011 to 0.69% in 2022 ( Table 4 ).

**Table 4:** Hospitalization and case-fatality rate of patients with MG, stratified per exacerbation and crisis

Year	Hospitalization	Deaths	Total MG patients		Case fatality rate							
					Exacerbation				Crisis			
					With		Without		With		Without	
			Rate	95% CI	Rate	95% CI	Rate	95% CI	Rate	95% CI	Rate	95% CI
**2010**	56	0	−	−	−	−	−	−	−	−	−	−
**2011**	263	2	0.76	± 1.05	0.76	± 1.05	−	−	0.85	± 1.67	0.68	± 1.34
**2012**	270	6	2.22	± 1.76	2.22	± 1.76	−	−	2.82	± 2.72	1.56	± 2.15
**2013**	323	2	0.62	± 0.86	0.62	± 0.86	−	−	1.34	± 1.85	0	± 0.0
**2014**	339	4	1.18	± 1.15	0.88	± 1.0	−	−	−	−	2.53	± 2.45
**2015**	342	4	1.17	± 1.14	0.88	± 0.99	−	−	1.16	± 1.6	1.18	± 1.62
**2016**	334	3	0.9	± 1.01	0.6	± 0.83	−	−	1.13	± 1.56	0.64	± 1.24
**2017**	378	6	1.59	± 1.26	1.32	± 1.15	−	−	1.84	± 1.79	1.24	± 1.71
**2018**	358	5	1.4	± 1.22	1.4	± 1.22	−	−	1.42	± 1.6	1.36	± 1.87
**2019**	355	8	2.25	± 1.54	1.97	± 1.45	−	−	2.04	± 1.98	2.52	± 2.43
**2020**	272	5	1.84	± 1.6	0.74	± 1.02	−	−	0.71	± 1.39	3.03	± 2.92
**2021**	240	14	5.83	± 2.97	4.58	± 2.65	−	−	3.05	± 2.95	9.17	± 5.42
**2022**	258	5	1.94	± 1.68	1.16	± 1.31	−	−	0.69	± 1.35	3.54	± 3.41
**2023**	210	4	1.9	± 1.85	1.43	± 1.6	−	−	−	−	4.6	± 4.4

Abbreviations: CI, confidence interval; MG, myasthenia gravis.

**Figure 1:**
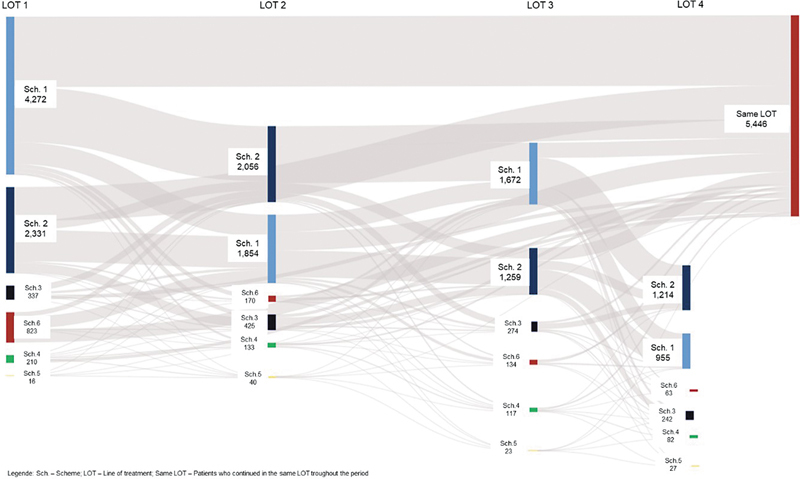
Abbreviations: Sch, scheme; LOT, line of treatment; Same LOT, patients who continued in the same LOT throughout the period. Sankey diagram of treatment patterns per scheme for total cohort.

Regarding treatment patterns, data from 8,029 patients were. Figure 1 illustrates the transitions between the predefined treatment schemes in Table 1 , with the first and second schemes being the most common. There was notable movement between all schemes from the first LOT (LOT 1) to the second (LOT 2), particularly between the first and second schemes. Additionally, some patients remained on their initial treatment scheme throughout the study period ( Figure 1 ).

**Figure 2:**
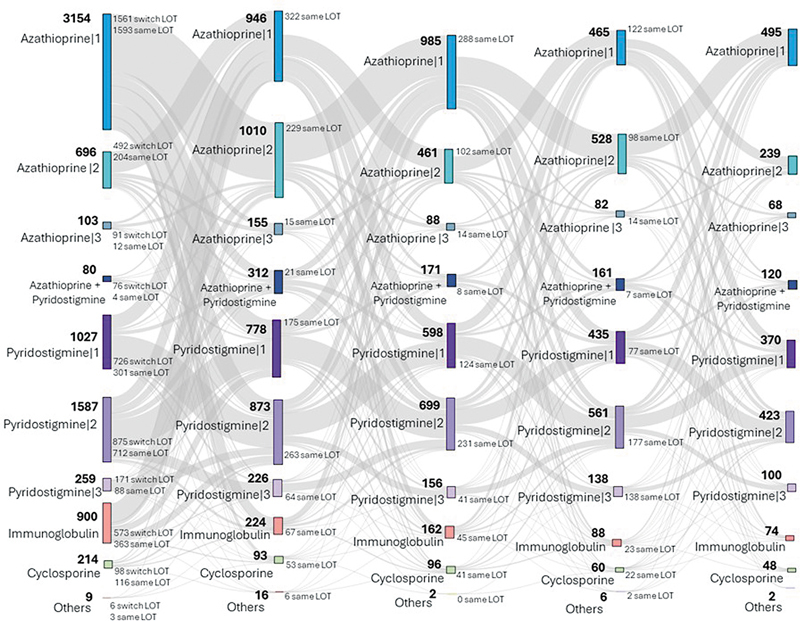
Abbreviations: LOT, line of treatment; Same LOT, patients who started the LOT and continued the same treatment up to the last FUP. Notes: Azathiprine 1: < 200 mg; 2: ≥ 200 mg; 3: ≥ 300 mg. Pyridostigmine 1: < 240 mg; 2: ≥ 240 to < 420 mg; 3: ≥ 420 mg. Azathioprine + pyridostigmine concomitance includes all the combination dosages found. Cyclosporine combines dosages < 350 mg and ≥ 350 mg. Immunoglobulin combines immunoglobulin only and immunoglobulin plus other medicaments. Others: Other medicaments and combinations. Sankey diagram of treatment patterns per LOT for total cohort.

The average number of LOT switches per patient increased as the FUP years progressed ( **Supplementary Table S1** , online only http://www.datasus.gov.br ). Figure 2 illustrates frequent transitions, showing both increases and decreases in medication dosages and the use of concomitant treatments. Reinforcing the severity of disease, IVIg was administered to patients alongside all other medications and dosages across all treatment lines.

Azathioprine and pyridostigmine were the most used medications for treatment. In LOT 1, some patients received medications with different dosages and used more than one advanced treatment concomitantly. The most common treatments in LOT 1 were azathioprine < 200 mg (39.3%), pyridostigmine between 240 and 420 mg (19.8%), pyridostigmine < 240 mg (12.8%), and azathioprine between 200 and 300 mg (8.7%), as shown in Figure 2 and **Table S2** (online only http://www.datasus.gov.br ). It is important to highlight that the 1,593 patients who started the first LOT with azathioprine < 200 mg did not switch LOT over the years ( Figure 2 ). The frequency and proportion of each treatment and combination are described in **Table S2** (online only http://www.datasus.gov.br ).

Switches of LOT occurred mainly between azathioprine < 200 mg and ≥ 200 to < 300 mg, as well as pyridostigmine < 240 mg and ≥ 240 to < 420mg. In the third LOT, it is possible to see an increase in cyclosporine use (2.6%), as shown in **Table S2** (online only http://www.datasus.gov.br ). It is important to highlight that a significant number of the patients who started treatment with cyclosporine did not switch treatment throughout the years ( Figure 2 ).

**Figure 3:**
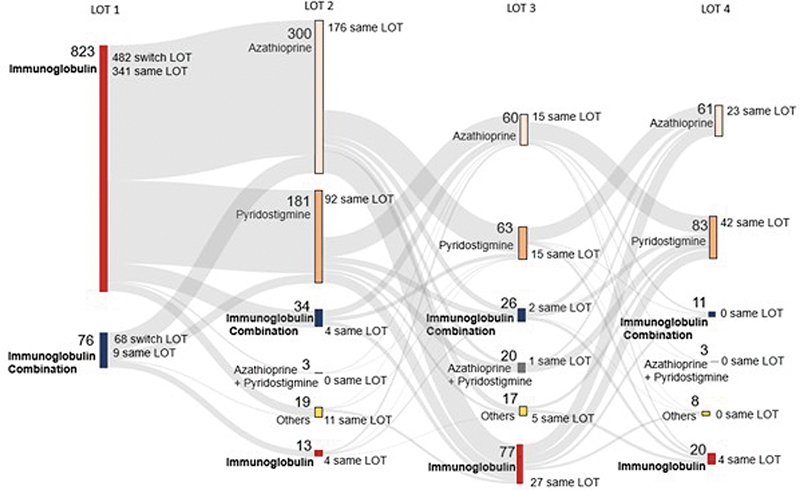
Abbreviations: LOT, line of treatment; Same LOT, patients who started the LOT and continued the same treatment up to last FUP. Notes: Each medication demonstrated includes all the combination dosage. Immunoglobulin combination: immunoglobulin plus other medications. Others: Other medicaments and combinations. Sankey diagram of treatment patterns per LOT for patients who started treatment with IVIg.

In 11% (n = 899) of MG patients the initial treatment was IVIg, with 823 using it alone and 76 using it in combination with other medications. Some patients switched to other treatments but returned to IVIg in subsequent LOTs. Additionally, some patients began treatment with IVIg, and continued throughout the study period, highlighting the severity of the disease, as shown in Figure 3 and **Table S3** (online only http://www.datasus.gov.br ).

In our MG cohort, the mean (SD) use of IVIg per patient per year was 4.2 (3.2). Recognizing that patients transitioned between treatment schemes ( Table 1 ), we assessed the number of IVIg uses per year based on the highest scheme reached ( Table 5 ). The mean (SD) use of IVIg among patients who advanced to scheme 5 was 4.2 uses per year. For patients who only used IVIg, the mean (SD) use was 4.8 (3.5) per year.

**Table 5:** Immunoglobulin use, outpatients and inpatients admission of patients with MG according to the maximum scheme patient advanced

		Patients by maximum scheme advanced					
	All patients	1	2	3	4	5	IVIg only
**IVIg use PPPY**							
**n**	8,029	2,447	3,520	1,213	380	128	341
**Mean (SD)**	4.2 (3.2)	4.4 (3.4)	3.6 (2.7)	4.1 (3.3)	3.8 (2.8)	4.2 (3.2)	4.8 (3.5)
**Median (min** – **max)**	3.0 (1.0–15.0)	3.0 (1.0–14.0)	3.0 (1.0–13.0)	3.0 (1.0–13.0)	3.0 (1.0–12.0)	3.0 (1.0–12.0)	3.0 (1.0–15.0)
**IQR**	4	4	3	5	3.5	4	3
**Outpatient visits PPPY**							
**n**	8,025	2,447	3,520	1,213	380	128	341
**Mean (SD)**	8.3 (4.8)	7.7 (3.8)	8.2 (3.7)	8.9 (6.4)	9.3 (7.7)	9.3 (7.0)	5.0 (4.2)
**Median (min–max)**	9.0 (1.0–166.0)	9.0 (1.0–47.0)	9.0 (1.0–48.0)	9.0 (1.0–166.0)	9.0 (1.0–114.0)	9.0 (1.0–76.0)	3.0 (1.0–48.0)
**IQR**	6	8	6	6	6	6	3
**Hospital admissions PPPY**							
**n**	482	124	196	91	28	15	28
**Mean (SD)**	1.5 (1.2)	1.5 (1.5)	1.4 (1.0)	1.5 (1.0)	1.8 (1.4)	1.3 (0.7)	1.4 (0.8)
**Median (min–max)**	1.0 (1.0–14.0)	1.0 (1.0–14.0)	1.0 (1.0–8.0)	1.0 (1.0–6.0)	1.0 (1.0–7.0)	1.0 (1.0–4.0)	1.0 (1.0–4.0)
**IQR**	0	0	0	1	1	0	0.25

Abbreviations: IQR, interquartile range; IVIg, intravenous immunoglobulin; MG, myasthenia gravis; PPPY, per patient per year; SD, standard deviation.

Among patients in treatment, 8,025 patients had an admission in the ambulatory setting. The mean (SD) ambulatory visit was 8.3 (4.8) per patient per year. Based on the highest scheme each patient reached, those who advanced to scheme 5 had a median of 9.3 (7.0) ambulatory admissions per year. Regarding hospitalizations > 24 hours, 482 patients who were treated had a hospital admission, with a mean (SD) of 1.5 (1.2) per patient per year ( Table 5 ).

## DISCUSSION

The present study provides a real-world descriptive assessment of treatment patterns for MG in the Brazilian Public Healthcare System. The treatment journey of MG patients shows efforts to control the disease, followed by periods of progression indicating inadequate symptom control and possible refractory disease. This involves frequent changes in medications, dosages, concomitant treatments, and the use of advanced medications, like IVIg. This study highlights the significant impact on patients' quality of life and functional status. Given the limited MG national data in Brazil, this is the first study to describe the treatment journey of this cohort in the country, aiming to fill this knowledge gap.

Aligned with published studies that have demonstrated a steady upward trend in MG cases over the past half-century, 19 20 an increase in admission of patients with MG to the Brazilin public healthcare system was noted in this study. The rise in MG diagnosis is largely attributed to advances in detection, treatment, and an overall increase in life expectancy. 19 21 However, despite identifying a total 13,476 patients with MG from 2010 to 2023, challenges in diagnosing the disease remain, which may lead to underreporting cases. 21

In our sample, patients who presented an exacerbation (30.4%) and crisis (9.4%) also had an increase in admission in healthcare units over the years, illustrating the potential severity of MG. This may also reflect an increased awareness of this disease among healthcare professionals and patients, as well as improvements in access to diagnosis and SUS services. The average age of patients experiencing exacerbations and crises was lower than the average age of the entire cohort. It may be because more than 65% of patients with exacerbation were female, and the literature indicates that MG primarily affects younger women (< 40 years) and older men (> 60 years). 8

Given the dynamic of the disease, the treatment pattern for patients with MG in this study was marked by medication switches, adjustments in dosage, and concomitant treatments, reinforcing the disease's constant stabilization and advancement of severity. The number of LOT switches per patient increased in the longer FUPs, reinforcing the difficulty in management.

As in LOT 1, most patients were treated with azathioprine and pyridostigmine, which may reflect limited access to more modern therapies or indicate undertreatment of patients with refractory forms of the disease, contributing to the higher frequency of exacerbations and ICU admissions. A recent study 22 demonstrated that mycophenolate mofetil has greater clinical efficacy and a better tolerability profile compared to azathioprine, suggesting that expanding access to alternative immunosuppressants may be essential to improve clinical outcomes in MG patients in the SUS.

The severity of the disease is underscored by patients using IVIg as a primary LOT, which is considered the most advanced treatment according to the Brazilian PCDT. This treatment was also used in concomitance with other medications across all LOTs. The mean use among patients in advanced schemes was more than 4 times per year. Some patients required continuous use of this medication, with a mean use of almost 5 times per year, highlighting the gravity and lack of stabilization of the disease, which directly impacts their quality of life. These results align with a real-world study from the United States, 23 which showed that half of the patients with gMG received multiple IVIg courses within the 1 ^st^ year of treatment, indicating higher than expected usage. Despite its effectiveness and generally well-tolerated use, continuous use has been associated with adverse events. 24

The ongoing health assistance required by MG patients 20 is highlighted by this study, which shows that patients visit ambulatory care an average of 8.3 times per year and have a mean number of hospitalizations of 1.5 times per year. Deaths related to MG highlight the challenges in managing the disease's severity. 17 25 The reduction in mortality trends has been attributed to improvements in diagnosis, immunosuppressive treatments, and intensive care for patients in myasthenic crisis. 17 25 In this study, the CFR increased from 0.76% in 2011 to 1.90% in 2023. This increase may be due to less misreporting and more patient admissions to the healthcare system. 26 Deaths only occurred among patients who had an exacerbation, and were higher among those who experienced a crisis.

The increase in the fatality rate observed in recent years may have been influenced by external factors, such as the COVID-19 pandemic. A study 27 conducted with 53 MG patients reported that, among the 9 unvaccinated patients who contracted COVID-19, 4 (44%) died, while no serious adverse events were observed among those vaccinated, even though 45% reported mild and self-limiting adverse effects after vaccination. These findings reinforce the importance of vaccination and continuous monitoring of these patients, especially in health crises.

Although the present study did not directly assess the presence of comorbidities, it is important to recognize that these conditions can significantly influence the severity of MG and clinical outcomes, including hospitalizations and mortality. A previous Italian multicenter study 28 with 178 hospitalized patients has demonstrated a high prevalence of cardiovascular, autoimmune, and neoplastic comorbidities in patients with MG, reinforcing the complexity of clinical management in such cases.

The present study has limitations, including incomplete and nonmandatory data intrinsic to retrospective studies. The administrative and reimbursement-focused databases lack detailed clinical data. Another important limitation is the lack of data on the use of oral corticosteroids, such as prednisone, which are widely used as first-line treatment. As a consequence, the treatment patterns described in this study predominantly reflect patients in more advanced stages of the disease, who require additional immunosuppressive therapies or continuous use of IVIg. Thus, this study mainly reflects severe cases and patients in crisis, potentially overestimating these groups while underestimating milder ones treated at home. Misdiagnosis or underreporting may also lead to underestimation.

This study provides a nationally comprehensive and detailed analysis of the patient journey and current treatment practice, highlighting that dynamic course of MG and the challenges of disease management. The fluctuations between drugs, dosage adjustments and the addition of medications reflect the individualized nature of treating this disease, which is often tailored to the patient's response to therapy. We identified areas for improvement and modification, ultimately aiming to enhance patient care and contribute valuable data to the field. These insights can help healthcare professionals better understand patients' needs and develop more effective treatment strategies.
