# Erratum

**DOI:** 10.1055/s-0045-1815745

**Published:** 2026-05-12

In the article “Patient journey and treatment pattern in myasthenia gravis: real-world data from the Brazilian public health system”, published in the Arq Neuropsiquiatr 2025; 83(09):s00451811720 under DOI: 10.1055/s-0045-1811720 .

**Where it was written:**

Review Article

**Should be:**

Original Article

